# A tree species classification model based on improved YOLOv7 for shelterbelts

**DOI:** 10.3389/fpls.2023.1265025

**Published:** 2024-01-18

**Authors:** Yihao Liu, Qingzhan Zhao, Xuewen Wang, Yuhao Sheng, Wenzhong Tian, Yuanyuan Ren

**Affiliations:** ^1^ College of Information Science and Technology, Shihezi University, Shihezi, China; ^2^ Geospatial Information Engineering Research Center, Xinjiang Production and Construction Corps, Shihezi, China; ^3^ Hubei Subsurface Multi-scale Imaging Key Laboratory, School of Geophysics and Geomatics, China University of Geosciences, Wuhan, China; ^4^ College of Mechanical and Electrical Engineering, Shihezi University, Shihezi, China

**Keywords:** unmanned aerial vehicle (UAV), tree species classification, image recognition, YOLO series algorithms, coordconv, attention mechanism

## Abstract

Tree species classification within shelterbelts is crucial for shelterbelt management. The large-scale satellite-based and low-altitude drone-based approaches serve as powerful tools for forest monitoring, especially in tree species classification. However, these methods face challenges in distinguishing individual tree species within complex backgrounds. Additionally, the mixed growth of trees within protective forest suffers from similar crown size among different tree species. The complex background of the shelterbelts negatively impacts the accuracy of tree species classification. The You Only Look Once (YOLO) algorithm is widely used in the field of agriculture and forestry, ie., plant and fruit identification, pest and disease detection, and tree species classification in forestry. We proposed a YOLOv7-Kmeans++_CoordConv_CBAM (YOLOv7-KCC) model for tree species classification based on drone RGB remote sensing images. Firstly, we constructed a dataset for tree species in shelterbelts and adopted data augmentation methods to mitigate overfitting due to limited training data. Secondly, the K-means++ algorithm was employed to cluster anchor boxes in the dataset. Furthermore, to enhance the YOLOv7 backbone network’s Efficient Layer Aggregation Network (ELAN) module, we used Coordinate Convolution (CoordConv) replaced the ordinary 1×1 convolution. The Convolutional Block Attention Module (CBAM) was integrated into the Path Aggregation Network (PANet) structure to facilitate multiscale feature extraction and fusion, allowing the network to better capture and utilize crucial feature information. Experimental results showed that the YOLOv7-KCC model achieves a mean average precision@0.5 of 98.91%, outperforming the Faster RCNN-VGG16, Faster RCNN-Resnet50, SSD, YOLOv4, and YOLOv7 models by 5.71%, 11.75%, 5.97%, 7.86%, and 3.69%, respectively. The GFlops and Parameter values of the YOLOv7-KCC model stand at 105.07G and 143.7MB, representing an almost 5.6% increase in F1 metrics compared to YOLOv7. Therefore, the proposed YOLOv7-KCC model can effectively classify shelterbelt tree species, providing a scientific theoretical basis for shelterbelt management in Northwest China focusing on Xinjiang.

## 1 Introduction

Shelterbelts, encompassing both natural and artificial woodlands, play a vital role in sustaining environmental well-being by serving a multitude of functions. These include reducing wind velocity, suppressing dust emissions, and enhancing microclimatic conditions (Qiao et al., 2016). Moreover, they contribute to increased ground vegetation coverage, modify wind flow patterns, and improve internal air circulation within forested areas (Liu et al., 2020). The establishment of shelterbelts emerges as a pivotal strategy for safeguarding desert ecosystems, concurrently standing as the most prevalent and effective method for mitigating and controlling desertification. In the Xinjiang region, situated on the western border of China, these protective forests play a crucial role in alleviating ecological degradation in Xinjiang’s desert areas (Cheng et al., 2023).

To combat desertification of northern regions in China, the Chinese government initiated afforestation and reforestation plans in 1978, notably through the Three-North Shelter Forest Program (TNSFP) (Cao, 2008). The forest protection policies based on TNSFP have significantly contributed to the increase in forest cover in China (Viña et al., 2016; Hu et al., 2021). Managed primarily through mixed forests, protective forests enhance resistance to pests and diseases, thereby fortifying ecological stability (Nilsson et al., 2006). The precise and efficient identification of tree species within protective forests holds paramount significance for ensuring their sustainable management (Hościło and Lewandowska, 2019).

In the early stages, tree species classification relied on field surveys, employing visual methods to identify tree species based on external morphological features such as roots, stems, leaves, flowers, fruits, and seeds (Li et al., 2021). While this method accurately captures tree species information in specific regions, it is labor-intensive and costly. With the rapid advancement of Unmanned Aerial Vehicle (UAV) technology, high-resolution UAV images have gradually replaced traditional field surveys and found widespread applications in forestry (Aeberli et al., 2023). Wang B. et al. (2023) utilized UAV LiDAR and hyperspectral data in the Maoershan forest area, achieving a tree species classification accuracy exceeding 78% through machine learning algorithms. Raczko and Zagajewski (2017) employed support vector machine (SVM), random forest (RF), and neural network for the classification of the five most common tree species in the Szklarska Poręba region using airborne hyperspectral images. The results indicated an accuracy of 77% for the neural network classifier, 68% for SVM, and 62% for RF. However, these studies often face challenges in pixel-based segmentation, especially in areas with high woodland density, leading to inter-crown occlusion and incomplete detection of diminutive individual trees.

In recent years, the deep learning methods has bestowed upon tree species classification a novel and effective perspective. Object detection methods find extensive applications in forestry research. YOLO (You Only Look Once) stands as a frequently employed single-stage object detection algorithm, distinguished by its rapidity and high precision (Redmon et al., 2016). Since its inception by Redmon et al., 2016, researchers have continuously evolved its series, refining and devising new variants such as YOLOv2 (Redmon and Farhadi, 2017), YOLOv3 (Redmon and Farhadi, 2018), YOLOv4 (Bochkovskiy et al., 2020), YOLOv5 (Jocher et al., 2022), YOLOX (Ge et al., 2021), YOLOv6 (Li et al., 2022), etc. Safonova et al. (2022) conducted a comparative analysis of YOLOv2, YOLOv3, and YOLOv4, with YOLOv4 achieving an impressive mean average precision (mAP) of 95% in detecting small bark beetles in Norwegian spruce trees. Jintasuttisak et al. (2022) employed YOLOv5 to detect date palm trees through UAV imagery, achieving a recognition accuracy of 92.34%. These studies showcase the formidable performance of the YOLO series in the domain of forestry applications. YOLOv7, introduced by Wang C. et al. (2023), aims to enhance detection performance through improved network architecture and training strategies. Wu et al. (2022) refined the YOLOv7 model for swift detection of tea oil fruit in camera-captured images, yielding a recognition accuracy of 96.03%. Yuan (2023) conducted a comparative evaluation of YOLOv4 and YOLOv7 models in classifying apple buds under high-quality image annotation requirements. Due to the limited availability of training images, YOLOv7 attained an mAP of 80% at 100% image annotation quality and 63% at 5% image annotation quality. The YOLOv7 model demonstrated outstanding performance in object detection, surpassing older versions of the YOLO detection model series in terms of training speed and accuracy. At present, there is a paucity of research addressing the issue of imbalanced distribution of tree species samples, as machine learning models tend to favor categories with a higher quantity, thereby impacting the predictive accuracy of minority categories. Additionally, the challenge of achieving accurate tree species classification in protective forests under complex background conditions, including lighting differences in UAV data collection, remains a significant topic worthy of research.

To address these above challenges, this study aims to develop a swift and precise model for tree species classification in protective forests. Specifically, we employed two data enhancement methods— (ie, geometric transformation and color transformation) —to address the issue of mixed tree species in shelterbelts and the uneven distribution of samples. The study proposes an improved YOLOv7 network, namely YOLOv7-KCC. Firstly, the K-means++ algorithm is adopted to cluster anchor boxes for all tree species labels in the dataset. This helps alleviate concerns related to the undue concentration or dispersion of initial clustering centers, thereby improving the quality and stability of the clustering results and expediting the model’s convergence during the training process. Secondly, the Coordinate Convolution (CoordConv) replaces specific convolutional layers in the feature extraction network. This integration facilitates the addition of corresponding coordinate information of tree species crowns with analogous features into the primary network. Such augmentation enhances the model’s capability to extract effective features without the introduction of superfluous parameters, thereby increasing the accuracy of the detection module’s localization regression. Finally, the Convolutional Block Attention Module (CBAM) is introduced to amplify feature extraction capabilities, mitigating interference from crown adhesion, occlusion, and background noise. The YOLOv7-KCC method aims to enhance the overall performance of the model in accurately classifying and detecting tree species within protective forests, particularly in complex and varied background conditions.

## 2 Study area and dataset

### 2.1 Study area overview

**Figure 1:**
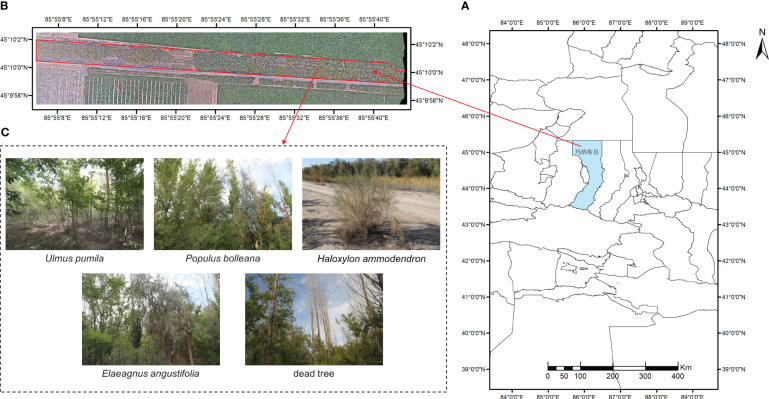
Schematic diagram of study area. **(A)** Topographic map of the study area; **(B)** Red underlined areas are the studied forest strips; **(C)** Details of tree species.

The study area is located at the northern foot of the Tianshan Mountains, in the southern part of the Junggar Basin, at the 150th regiment of the Moxowan Reclamation Area (Wang et al., 2022), Xinjiang Uygur Autonomous Region (45°10′N, 85°56′E, see **Figure 1**). Under the action of the northwest wind, the crescent-shaped sand dunes at the edge of the regiment are a typical windy landform, and the forest coverage of the regiment is 38%. The windbreak and sand-fixing shelterbelts are composed of a combination of tree species, including *Populus bolleana*, *Ulmus pumila*, *Elaeagnus angustifolia*, *Haloxylon ammodendron*, *Tamarix chinensis*, *Alhagi sparsifolia* and dead trees, with the aim of stabilizing sand dunes and protecting cultivated land. The *Ulmus pumila* and mixed broadleaf forests are distributed along both sides of the road. The vertical structure of the windbreak and sand-fixing shelterbelts includes a canopy layer, a shrub layer, and an herb layer. The primary focus of this paper is to investigate and classify tree species, including *Ulmus pumila*, *Elaeagnus angustifolia*, *Populus bolleana*, *Haloxylon ammodendron*, and dead trees. For dead trees, the relevant departments of forestry management will subsequently remove and replant them. Therefore, we have not specified the type, but have categorised it uniformly as dead trees.

### 2.2 Data acquisition

**Figure 2:**
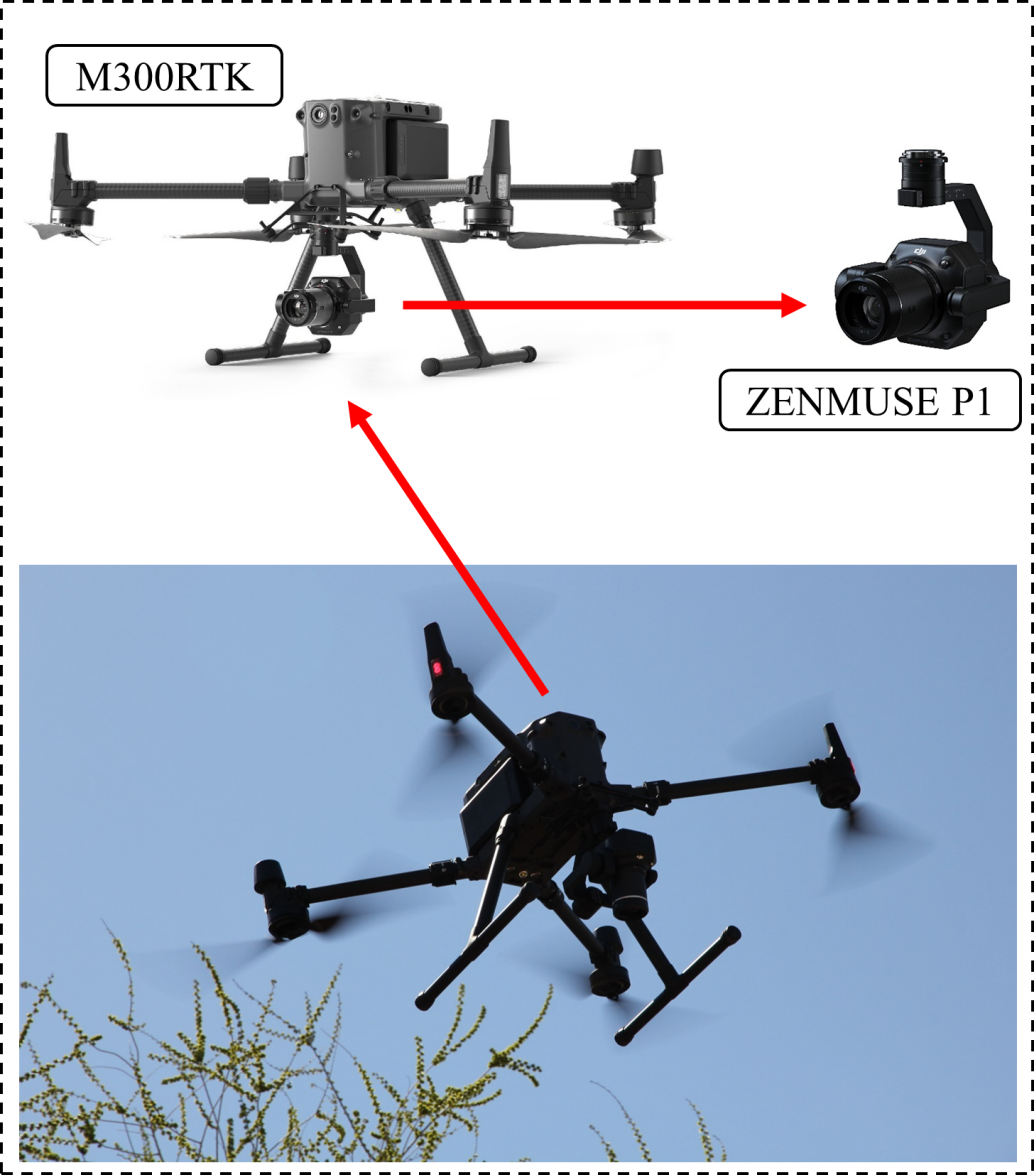
DJI M300 RTK UAV Platform.

In this study, the DJI M300RTK multi-rotor grade UAV is used, as shown in **Figure 2**. The RGB sensor employed is the DJI P1, a high-performance, multi-purpose aerial survey payload, equipped with a 45-megapixel full-frame image sensor. The sensor incorporates a DJI DL 35mm F2.8LS ASPH Lens with a focal length of 35mm, and the ground sample distance (GSD) and shooting distance (L) establish a relationship of GSD=L/80.

On August 1st, 2022, between 5:00 PM and 6:00 PM, experimental data were collected under cloudy weather conditions using the DJI M300RTK. This time period was chosen because between 1:00 PM and 2:00 PM that day had harsh sunlight, which resulted in poor image quality with excessive exposure, leading to numerous white spots and less distinct features in the tree canopy imagery. Therefore, the flight at 5:00 p.m. was selected when it was cloudy. The UAV flew at a relative altitude of 100m and a speed of 13.7m/s, with a ground sampling distance (GSD) of 1.25cm/pixel. The gimbal shooting angle was vertical to the ground, and the heading and side overlap rates were 75%. A total of 1003 RGB visible remote sensing images were obtained, each with dimensions of 8192 pixels × 5460 pixels. Following the acquisition of the UAV image data, a field survey of tree species information was conducted in the experimental area, which involved gathering and recording information on the latitude and longitude, sampling photos and tree species.

### 2.3 Data preprocessing and dataset construction

The images captured by UAVs and processed by the Pix4D Mapper software generate digital orthophoto map (DOM), digital surface models (DSM), and point cloud datasets. Aerial triangulation calculations are employed to generate the point cloud models, and the images are automatically calibrated to produce visible orthophotos and DSM images. The visible orthophotos obtained are stored in TIF file format. After non-forested areas, such as black edge fill, cotton fields, bare ground roads, and farmlands, are removed from the images, a Python script is used to randomly crop the TIF images, which are then saved in JPG format. The result is 396 images of the protective forest belt, each with a resolution of 640×640 pixels. The images are annotated using the labelImg tool in the Pascal VOC dataset labeling format (Shetty, 2016), which produces an XML label file containing information on the target location, anchor frame size, and labels for different tree species. To effectively train deep neural networks, a significant amount of data is required. Small datasets are prone to overfitting (Wu et al., 2022), which can compromise the robustness and generalization ability of neural network models (Chen et al., 2022). To mitigate this issue, data augmentation of the acquired data is necessary (Jia et al., 2017). In this study, we utilize the Python language to invoke the OpenCV image processing library (Gollapudi, 2019). This enables us to flip, rotate, adjust contrast, add gaussian noise, and apply other techniques to enhance the collected images. Furthermore, we perform synchronous transformation on the corresponding annotation file of each image, which significantly expands the sample set of images to 4356. These images are then randomly divided into a training set of 3048, a validation set of 872, and a test set of 436, according to a 7:2:1 ratio. The distribution and number of datasets are outlined in **Table 1**. Also, **Table 2** shows the number of different tree species in our dataset.

**Table 1:** The partitioning of the dataset.

	Name	Proportion	Number of Picture	Number of Trees
Dataset	Training Set	70%	3048	28113
	Validation Set	20%	872	7866
	Test Set	10%	436	4116
Total		100%	4356	40095

**Table 2:** The number of different tree species in the dataset.

Name	Ulmus pumila	Populus bolleana	Haloxylon ammodendron	Elaeagnus angustifolia	dead trees
Number of Trees	12573	4861	2371	13352	3025
Proportion	34.75%	13.43%	6.55%	36.90%	8.36%

Additionally, prior to feeding the dataset into the neural network model for training, 80% of the training set is randomly selected for Mosaic data augmentation, followed by random selection of 80% of the Mosaic-augmented training set for Mixup data augmentation. The Mosaic data augmentation method, proposed in YOLOv4 as an improvement to the CutMix data augmentation method (Bochkovskiy et al., 2020), involves random selection of four images, random scaling, and random distribution for splicing, to increase the number of targets in a single image and enrich the detection dataset. In particular, random scaling adds many small targets, improving the robustness of the network. Directly computing the data from four images reduces the required Mini-batch size and effectively reduces GPU memory usage (Bochkovskiy et al., 2020). MixUp is a data augmentation strategy based on mixing classes, allowing for the combination of images from different classes to expand the training dataset. In our model, the input training set is first enhanced with Mosaic data at a settable ratio, and then the images after being Mosaic enhanced are later enhanced with MixUp at a settable ratio.

## 3 Improved YOLOv7 tree species classification model design for shelterbelts

**Figure 3:**
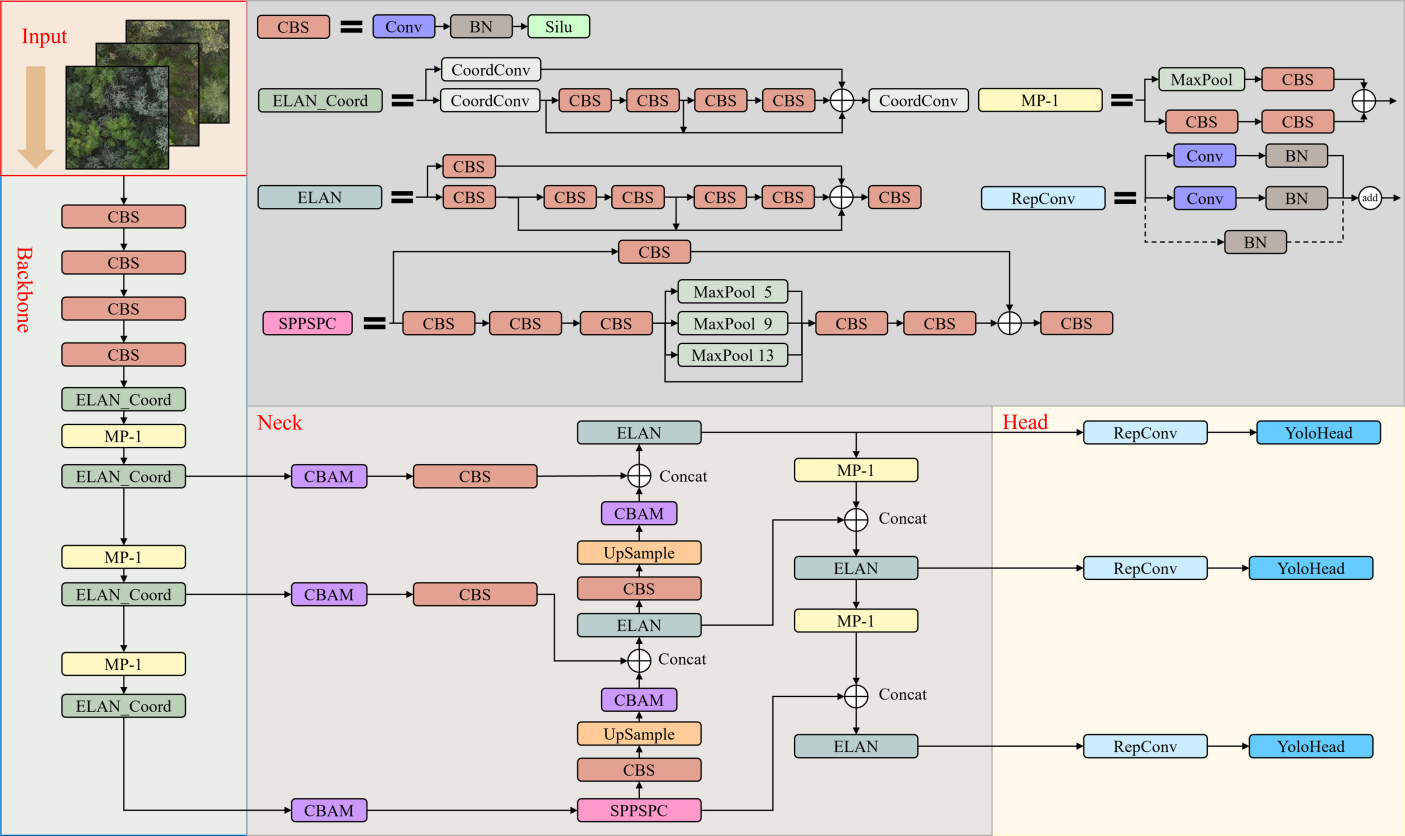
The architecture of the YOLOv7-KCC network.

Although YOLOv7 performs well in real-time object detection (Zhao et al., 2023), however its detection performance on small targets such as dead trees and *Haloxylon ammodendron*, which are affected by complex backgrounds, may fall short of expectations. Additionally, for large canopy trees such as *Elaeagnus angustifolia* and *Ulmus pumila*, crown overlap can result in false positives or misclassifications. To accurately identify small targets in complex backgrounds and precisely classify complex overlapping canopy species, we introduce in this section an improved YOLOv7-KCC model based on the native YOLOv7 network. **Figure 3** shows all the structures of the YOLOv7-KCC model, which consists of four parts: Input, Backbone, Neck, and Head. Additionally, we provide the composition structure of each module in detail.

The CBS module, comprises Convolution (Conv), Batch Normalization (BN), and the Silu activation function. Conv represents the convolutional layer, BN stands for the Batch Normalization layer, and Silu is an activation function. Additionally, ELAN_Coord is our novel module, an enhancement based on the Efficient Layer Aggregation Network (ELAN), which we will elaborate on in the subsequent sections, discussing the improvement concepts and implementation. MP-1 is an undersampling transition module. It consists of a combination of MaxPool and convolutional layers (Conv) to alter feature channel dimensions. MaxPool is a type of pooling operation, typically employed in Convolutional Neural Networks, aimed at reducing spatial dimensions and feature extraction. The CBAM module, denoting Convolutional Block Attention Module, will also be extensively detailed in forthcoming sections, including the rationale behind its inclusion and implementation. UpSample is an operation used for upscaling the spatial dimensions of images or feature maps. This operation corresponds to downscaling operations such as MaxPool and is employed to restore the resolution of images or feature maps to a higher level. SPPSPC is an abbreviation for Spatial Pyramid Pooling and Spatial Attention Module. SPPSPC combines two techniques. The Spatial Pyramid Pooling (SPP) is a pooling technique that enables the model to extract features at different scales, effectively adapting to objects of various sizes. The Spatial Attention Module (SPC) is an attention mechanism designed to enhance the model’s focus on regions of interest. RepConv consists of three branches. The uppermost branch comprises a 3×3 Convolution layer combined with BN (Batch Normalization) for feature extraction. The middle branch consists of a 1×1 Convolution layer with BN, intended for feature smoothing. The final branch is an Identity, not performing convolution operations, and directly passing through. YoloHead is an integral detection component within the model, responsible for converting convolutional feature maps into tangible object detection outcomes. This includes predictions of object positions and category labels.

### 3.1 Refining anchors via K-means++ clustering

The K-means algorithm, firstly proposed by Mac (1967), partitions a dataset into various clusters, it can maximize intra-cluster similarity while minimize inter-cluster similarity. Due to its simplicity and efficiency have led to widespread applications in various fields such as market analysis (Tleis et al., 2017), feature learning (Tang et al., 2017), document clustering (Sardar and Ansari, 2018), and image segmentation. In terms of object detection, anchor boxes play a crucial role as rectangular frames for predicting object positions and sizes. Traditionally, the number and dimensions of anchor boxes are manually configured, but this may not be the optimal choice. The initial anchor boxes in the YOLO series algorithm are obtained through k-means clustering on the MS COCO 2017 dataset (Lin et al., 2014). The COCO dataset comprises three parts, consisting of the COCO train-2017 training set, COCO val-2017 validation set, and COCO test-2017 test set. With over 33GB of images and instances of over 200,000 objects, it encompasses 80 categories of common everyday items. The K-means algorithm proves suitable for objective clustering in datasets with multiple categories or samples. However, these anchor boxes may not suit the tree species dataset in this study, emphasizing the importance of prudent anchor selection for improved position prediction accuracy.

The traditional K-means algorithm randomly select cluster center points, which can lead to convergence heavily dependent on the initialization of cluster centers. K-means++ has some improvements based on the K-means, it has follow steps: (a) randomly selecting a target box from the training set as the first cluster center point, (b) calculating the distance from each remaining target box to existing cluster center points and selecting the farthest target box as the next cluster center point, (c) repeating step (b) until all cluster center points are determined, (d) assigning all target boxes to the cluster to which the nearest cluster center point belongs, obtaining a collection of target boxes for each cluster, (e) assigning all target boxes to the cluster to which the nearest cluster center point belongs, obtaining a collection of target boxes for each cluster, and (f) returning the width and height of all anchor boxes as the final set of anchor boxes. One of the primary advantages of K-means++ is achieving better centroids in initial iterations, facilitating faster convergence of the entire algorithm. In terms of computational complexity, the initial centroid selection process in K-means++ is relatively more intricate compared to regular random selection. Therefore, on large datasets, the initial centroid selection may become time-consuming. Nevertheless, this overhead is typically offset throughout the iteration of the entire K-means algorithm.

**Figure 4:**
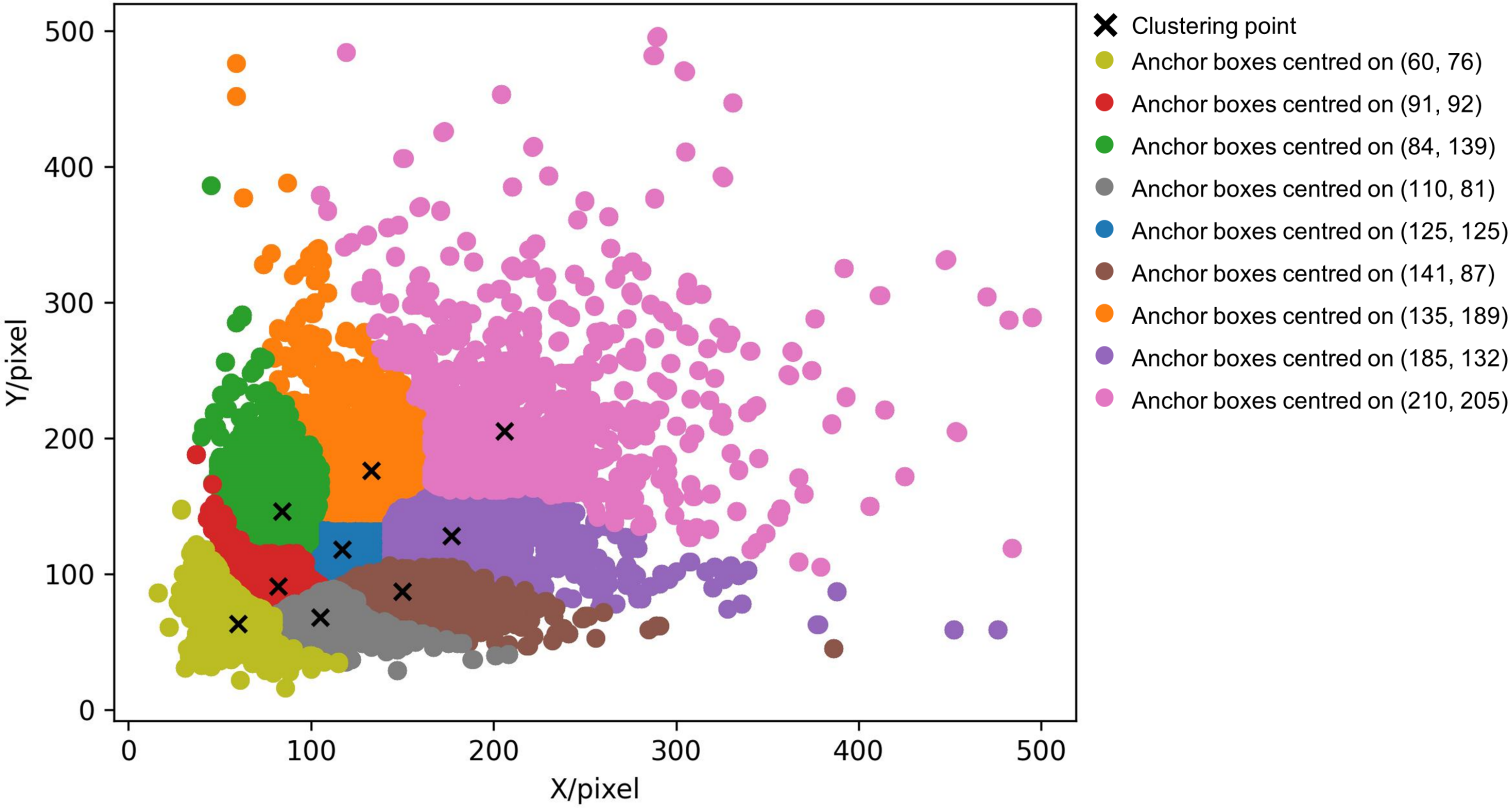
K-means++ clustering results with anchor boxes distribution.

The K-means++ algorithm is applied to our dataset, we obtained a more accurate and representative set of 9 anchor boxes: (60, 76), (110, 81), (91, 92), (84, 139), (141, 87), (125, 125), (185, 132), (135, 189), and (210, 205). **Figure 4** illustrates the clustering results. We employed the K-means++ algorithm on our dataset, partitioning the dataset into distinct clusters to facilitate the creation of a set of anchor boxes. These anchor boxes represent hypothetical bounding boxes, serving as reference points during the model’s training phase, aiding the model in learning how to discern and localize objects within images. The nine coordinates generated constitute the coordinates of these anchor boxes. During the training of our object detection model, these anchor boxes will be used to match with real object bounding boxes, determining which should be labeled as Positive Anchors (those with substantial overlap with real trees) or as Negative Anchors (those with minimal overlap with real trees). This process is instrumental in enabling the model to effectively predict object positions and categories.

### 3.2 Updates to the recognition module

The YOLOv7 object detection algorithm, introduced in July 2022 following continuous refinement of the YOLO series (Wang et al., 2022), maintains the exceptional speed, efficiency, durability, and precision. The sanctioned version of YOLOv7 exhibits a precision improvement of 120% and is 180% faster in terms of Frames Per Second (FPS) compared to YOLOv5, and 180% faster than YOLOX for equivalent volume (Wang et al., 2022). YOLOv7 shares similarities with its antecedents, it still employs CSPDarknet53 (Cross Stage Partial Darknet53) in the base network, known for enhancing accuracy, velocity, and superior feature expression capability. CSPDarknet53, a component of the YOLO family’s backbone network (Bochkovskiy et al., 2020), is an extended and improved version of the Darknet53 backbone network, designed to enhance model performance and efficiency. The Neck network employs the PANet (Path Aggregation Network) (Wang et al., 2019) path aggregation module, adept at aggregating features of varying scales, thereby improving the accuracy and robustness of target detection.

**Figure 5:**
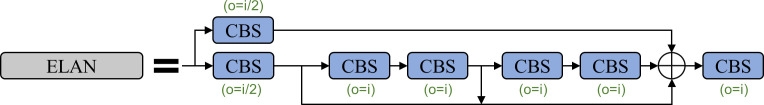
The structure of the ELAN module.

In comparison to the YOLOv5 network, YOLOv7 suggests the ELAN structure and the MP structure. ELAN, depicted in **Figure 5**, efficiently acquires more features by regulating the shortest and longest gradient paths. It encompasses two branches: 1) The first branch traverses a 1×1 convolution for channel number conversion, 2) the second branch first undergoes a 1×1 convolutional block for channel number conversion, then proceeds with four 3×3 convolutional modules for feature extraction. Based on the ELAN module, we shall obtain 2 feature maps exclusively processed by a single CBS module, 1 feature map derived from the treatment of three CBS modules, and 1 feature map generated through the processing of 5 CBS modules. Finally, these four feature layers will undergo another convolution-normalization-activation function (CBS) for feature integration. Such a dense stacking corresponds to a more intricate residual structure. Residual networks are characterized by their ease of optimization and the ability to significantly improve accuracy by increasing depth. The internal residual blocks utilize skip connections, mitigating the vanishing gradient problem associated with deep neural networks.

**Figure 6:**
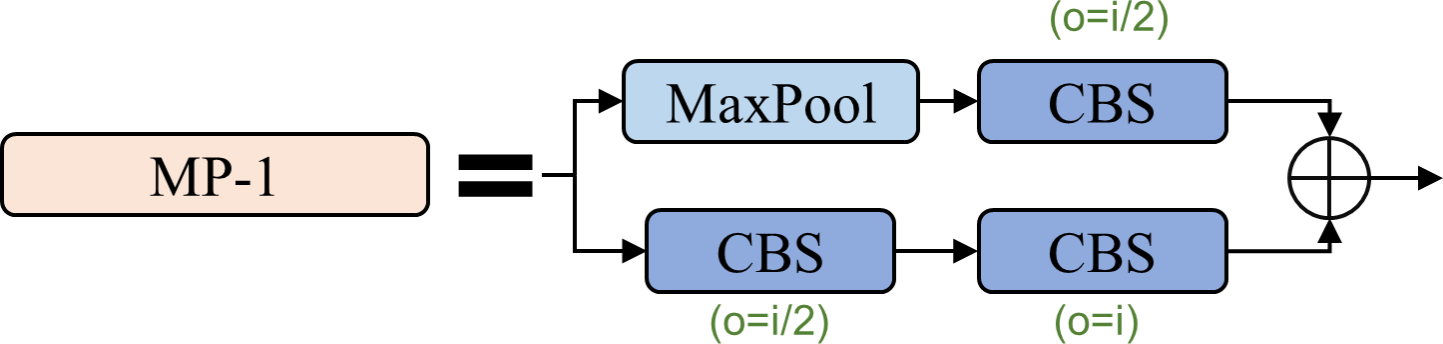
The structure of the MP.

The MP structure, illustrated in **Figure 6**, facilitates downsampling through two branches. The first branch employs max pooling for downsampling and a subsequent 1×1 convolution for channel modification. The second branch utilizes a 1×1 convolutional operation followed by a 3×3 convolutional block with a stride of 2 for downsampling. The results from both branches are combined to obtain a more deeply downsampled outcome.

**Figure 7:**
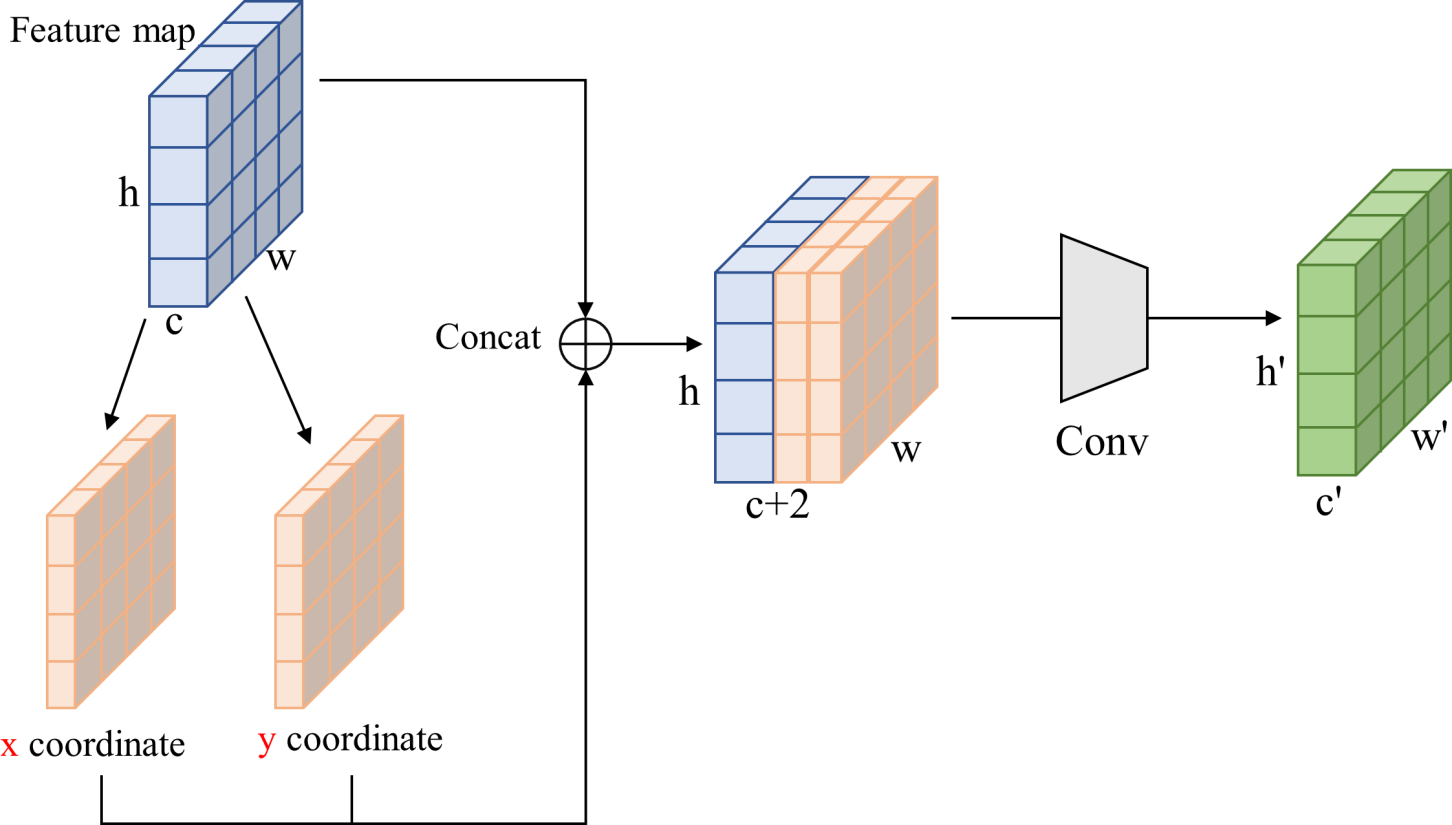
The structure of CoordConv convolution.

The dataset of protective forest tree species comprises a mere 4,356 images of 640×640 pixels, which is relatively diminutive in scale and predisposed to overfitting during training. To address this, the CBS module in the ELAN structure was replaced with the CoordConv module (Liu et al., 2018), as depicted in **Figure 7**. CoordConv incorporates positional information into the input feature map, enhancing the convolutional layer’s ability to discern pixel position information. This modification, replacing the ordinary 1×1 convolution in the ELAN module with CoordConv, allows the model to better capture positional information, thereby improving overall performance.

Through the substitution of the 1×1 convolution in the ELAN module with CoordConv convolution, the positional information from the input is conveyed to the subsequent convolution operation alongside the feature map. This enhancement renders the model more attuned to positional information, thereby elevating its sensitivity and overall performance.

### 3.3 Convolutional attention module

**Figure 8:**
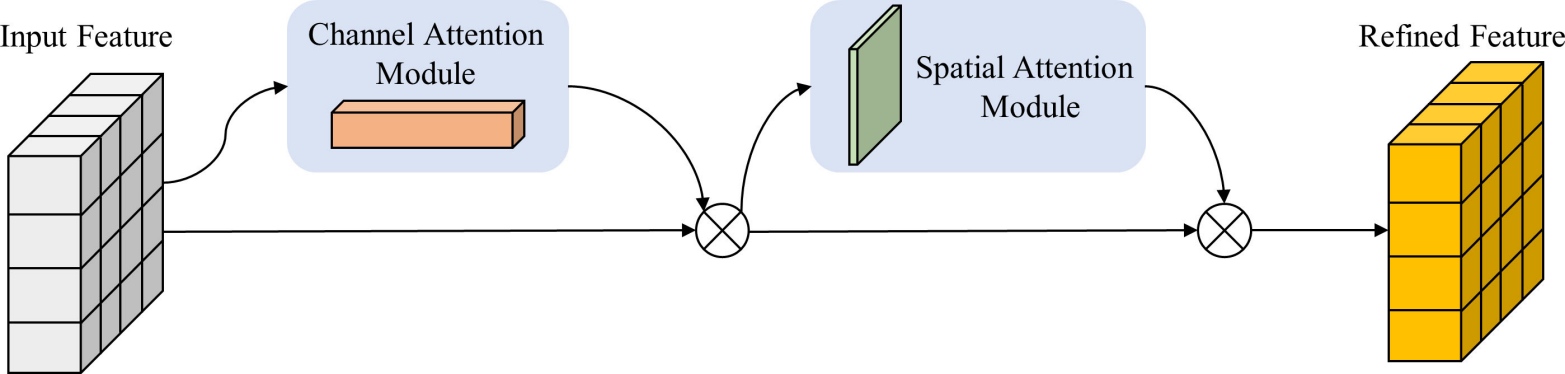
The structure of the CBAM Attention Module.

In convolutional neural networks, attention mechanisms have emerged as a pivotal technique, bestowing the network with enhanced discernment of vital features within the input, thereby augmenting overall performance of the network. The Convolutional Block Attention Module (CBAM), an attention mechanism deployed in convolutional neural networks, empowers the network to heighten its perceptual capability towards crucial features while attenuating noise and irrelevant information interference, thus elevating the performance of the network (Woo et al., 2018). It is composed of two sub-modules, the Channel Attention Module and the Spatial Attention Module, connected in series, as illustrated in **Figure 8**.

The Channel Attention Module endeavors to apportion weights to every channel of the input feature map to accentuate channels that are more pertinent to the task. It produces channel attention weights by means of a weighted fusion of the feature maps resulting from global average pooling (GAP) (**Equation 1**) and global maximum pooling (GMP) (**Equation 2**). The calculation formula for the channel attention weights is as follows:


(1) $$F_{GAP}\left(x_{c}\right)=\frac{1}{H\times W}\sum_{i=1}^{H}\sum_{j=1}^{W}x_{c}\left(i,j\right)$$

(2) $$F_{GMP}\left(x_{c}\right)=\max_{i=1,j=1}^{H,W}x_{c}\left(i,j\right)$$

In this context, $x_{c}$ refers to the *c*-th channel of the input feature map, while *H* and *W* respectively represent the height and width of the feature map. Subsequently, two fully connected layers (*FC*) and an activation function, such as ReLU, are employed to generate the channel attention weights (**Equation 3**).


(3) $$M_{c}=\text{σ}\left(\alpha F_{FC}\left(F_{GAP}\left(x_{c}\right)\right)+\beta F_{FC}\left(F_{GMP}\left(x_{c}\right)\right)\right)$$

The channel attention weights, $M_{c}$ , are calculated using the following formula, where *σ* represents the Sigmoid activation function and *α* and *β* are trainable parameters. Finally, the channel attention weights that have been calculated are applied to each channel of the input feature map, resulting in the output feature map, (**Equation 4**)


(4) $$y_{c}=M_{c}\cdot x_{c}$$

The Spatial Attention Module aims to allocate weights to each position of the input feature map, so as to focus on spatial regions that are more relevant to the task at hand. Firstly, the maximum (**Equation 5**) and average (**Equation 6**) values are computed for each feature point of the feature layer that has already been processed by the Channel Attention mechanism. Subsequently, these two results are stacked and the spatial attention weights are calculated through a convolutional layer. The formula for computing the spatial attention weights is as follows:


(5) $$F_{avg}\left(x\right)=\frac{1}{C}\sum_{c=1}^{C}x_{c}$$

(6) $$F_{max}\left(x\right)=\max_{c=1}^{C}x_{c}$$

Here, *C* denotes the number of channels in the input feature map. Subsequently, the per-channel average and per-channel maximum results are added, and the spatial attention weights are generated through a convolutional layer. The spatial attention weights (**Equation 7**):


(7) $$S=F_{conv}\left(F_{avg}\left(x\right)+F_{max}\left(x\right)\right)$$

Where $F_{conv}$ denotes a convolution operation with a 7×7 kernel. Finally, the computed spatial attention weights are applied to the input feature map, resulting in the output feature map (**Equation 8**):


(8) y = S☉x

Where ☉ represents element-wise multiplication.

In the ELAN module of our Backbone network, a 1×1 conventional convolutional CBS is employed, and the CBAM attention mechanism is integrated into the Neck network. The Backbone feature extraction network obtains three effective feature layers, denoted as feat1, feat2, and feat3. Before passing them into the enhanced feature extraction network for FPN construction, they are fed into the CBAM module to automatically learn the correlations and importance among the feature channels, resulting in weighted feature maps that are subsequently passed into the FPN network for convolutional operations. Additionally, the CBAM module is applied to the feature maps of the two upsampling layers in the FPN to further enhance the model’s expressiveness and detection performance.

## 4 Experimental results

### 4.1 Computer environment and parameter settings

The models were trained on a server configured with Intel (R) Xeon CPU, GeForce RTX 2080Ti 11GB GPU, Python 3.7 software environment and Pytorch 1. 8. 1 deep learning framework. The experimental parameters were set as follows. In the training process, we used the Adam optimizer without freezing the backbone, we set 300 iteration cycles (Epoch), the initial learning rate was 0.01, the weight decay was 0.0001, the learning rate momentum was 0.937, the learning rate descent method was cosine annealing algorithm (COS), the batch size was 4, the non-maximum suppression (NMS) threshold was 0.3, and the confidence threshold was 0.3. The confidence threshold is 0.5.

### 4.2 The performance evaluation metrics of the network model

For the study of tree species classification in complex environments, the accuracy and generalization ability of the detection network are taken into consideration. This study employs precision (**Equation 9**), recall (**Equation 10**), F1 score (**Equation 11**), and mAP (**Equations 12**, **13**) as evaluation metrics for the detection accuracy of the model. They are calculated using the following equation:


(9) $$Precision=\frac{TP}{TP+FP}\times 100\%$$

(10) $$Recall=\frac{TP}{TP+FN}\times 100\%$$

(11) $$F1=\frac{2\times \;Precision\;\times Recall\;}{\;Precision\;+\;Recall\;}$$

(12) $$AP=\int_{0}^{1}P\left(R\right)dR$$

(13) $$mAP=\frac{1}{M}\sum_{k=1}^{M}AP\left(k\right)\times 100\%$$

where, TP (True Positive) represents the number of correctly detected positive samples, which refers to predicted boxes with the same class as the labeled boxes and the Intersection over Union (IoU) greater than 0.5. FP (False Positive) represents the number of incorrectly detected positive samples, while FN represents the number of incorrectly detected negative samples. Precision and recall can be used to obtain evaluation metrics such as mAP@0.5 and F1@0.5. Where the “@” symbol in @0.5 indicates a specific threshold. @0.5 means using an IoU threshold of 0.5 for calculation. This is the main metric used in this study to measure the performance of the object detection model.

### 4.3 Ablation experiments

To evaluate the efficacy and feasibility of the proposed model, we conducted ablation experiments to scrutinize the impact of different components on the network’s performance. Using YOLOv7 as the baseline model, we investigated the influence of three enhancement methods. **Table 3** presents the results of our ablation experiments on the protective forest tree species dataset.

**Table 3:** Improved YOLOv7 ablation experiments.

Model	Precision	Recall	F1 Score	mAP@0.5	mAP@0.75
YOLOv7	94.94%	90.18%	0.924	95.22%	78.72%
YOLOv7_Kmeans++	94.50%	92.74%	0.936	96.45%	79.87%
YOLOv7_CoordConv	94.76%	91.92%	0.934	96.24%	80.33%
YOLOv7_CBAM	94.61%	93.31%	0.938	96.86%	81.52%
YOLOv7_ Kmeans++_CBAM	94.98%	93.81%	0.942	97.40%	82.58%
YOLOv7_ CoordConv _CBAM	95.86%	94.47%	0.952	97.50%	85.66%
YOLOv7_Kmeans++_CoordConv_CBAM	97.93%	98.12%	0.98	98.91%	92.92%

As indicated in the table, we re-clustered anchor boxes of the dataset using the K-means++ algorithm before model training. With the 9 newly obtained pre-trained anchor boxes, our model outperformed the native network on all 5 metrics. Substituting the ordinary convolutions in the ELAN module with CoordConv in YOLOv7 resulted in a 1.02% increase in mAP@0.5 and a 1.61% increase in mAP@0.75, underscoring the efficacy of CoordConv convolution in enhancing the detection accuracy and precision. Introducing the CBAM attention module to the Neck network layer, the model shows significant improvements in Recall and mAP, with Recall increasing by 3.13% and mAP@0.75 increasing by 2.80%. Thus, the model with added attention mechanism exhibited notable enhancement on all 5 metrics, effectively filtering out irrelevant information during feature extraction, prioritizing valid information extraction, and focusing more on learning target features. Furthermore, the combination of modules, as shown in the table, the CoordConv+CBAM yielded the highest accuracy improvement, achieving an F1 Score of 0.952—an excellent result. Ultimately, after re-clustering the anchor boxes using K-means++, replacing the main network with CoordConv convolution, and adding the CBAM attention module for feature extraction in the Neck network, the precision reached 97.93%. This marked a 2.99% improvement over the native YOLOv7 network, and the mAP@0.5 increased by 3.69%. The K-means++ re-clustering of anchor boxes optimized their selection, aligning them more closely with the dataset’s features, thereby improving accuracy and recall while reducing false positives and false negatives. The combination of CoordConv convolution with the CBAM attention module strengthened the learning of distinctive features expressed by various tree species in the protective forest dataset, significantly boosting accuracy.

**Figure 9:**
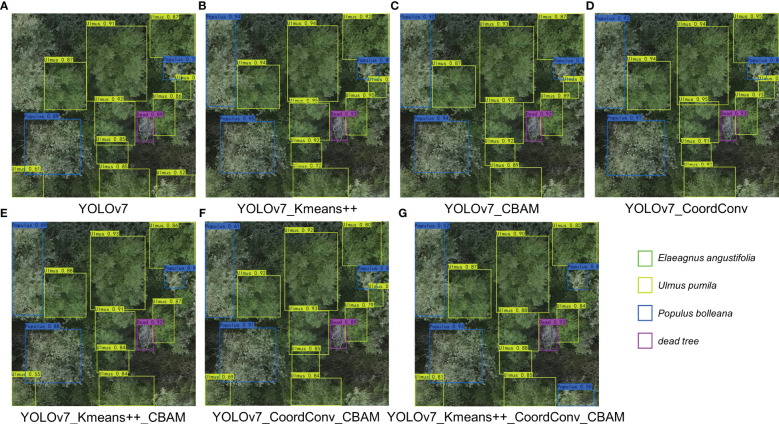
Result diagram of the improved model. **(A-G)** represent the results of YOLOv7, YOLOv7_Kmeans++, YOLOv7_CBAM, YOLOv7_CoordConv, YOLOv7_Kmeans++_CBAM, YOLOv7_CoordConv_CBAM, YOLOv7_Kmeans++_CoordConv_CBAM, respectively.

**Figure 9** elucidates the impact of various refinement strategies on the taxonomic classification of tree species in ablation experiments. The inherent YOLOv7 network exhibits a tendency to omit the classification of trees with partial crowns and conspicuous features, exemplified by the conspicuous absence of a *Populus bolleana* in the top-left corner of **Figure 9A**. The incorporation of CBAM attention mechanisms and CoordConv convolutional layers enhances the model’s capacity for feature extraction, thereby mitigating the prospect of oversights. In **Figure 9A**, a *Populus bolleana* erroneously classified as *Ulmus pumila* in the bottom-right corner attests to the model’s initial limitations. Nevertheless, following refinements, our YOLOv7-KCC adeptly rectifies such misclassifications.

### 4.4 Comparison of detection performance with other models

**Figure 10:**
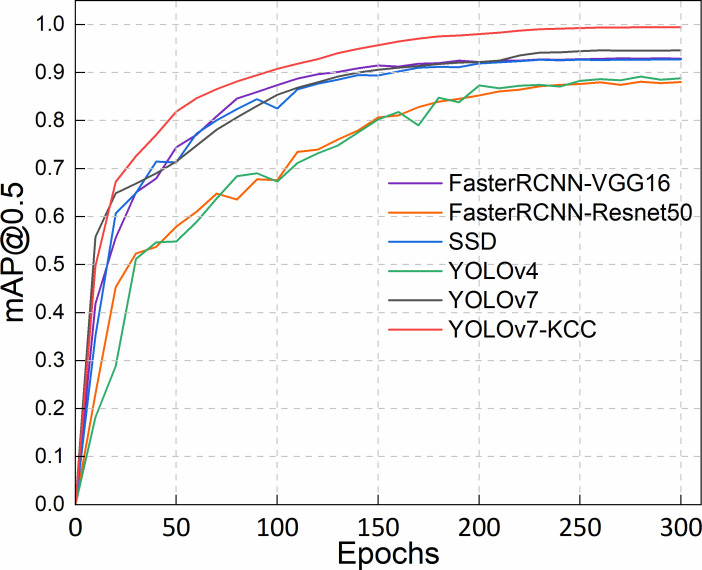
Accuracy variation of five object detectors.

To comprehensively assess the performance of the model proposed in this paper, including detection accuracy and model size on the protective forest tree species dataset, we compared our improved model with 5 state-of-the-art object detectors: FasterRCNN-VGG16, FasterRCNN-Resnet50, SSD, YOLOv4, and the baseline network YOLOv7. We plotted a line graph using mAP@0.5 as the metric. As shown in **Figure 10**, the model training accuracy graph demonstrates that YOLOv7 has a certain advantage in tree species classification. In the first 200 epochs, the recognition accuracy of Faster RCNN-VGG16, SSD, and YOLOv7 steadily improved and surpassed that of Faster RCNN-Resnet50 and YOLOv4. Beyond 200 epochs, the training accuracy of YOLOv7 outpaced SSD and Faster RCNN-VGG16. Notably, YOLOv7-KCC, enhanced based on YOLOv7, consistently demonstrated superior performance, exhibiting smoother accuracy curves and higher accuracy compared to other models.

**Figure 11:**
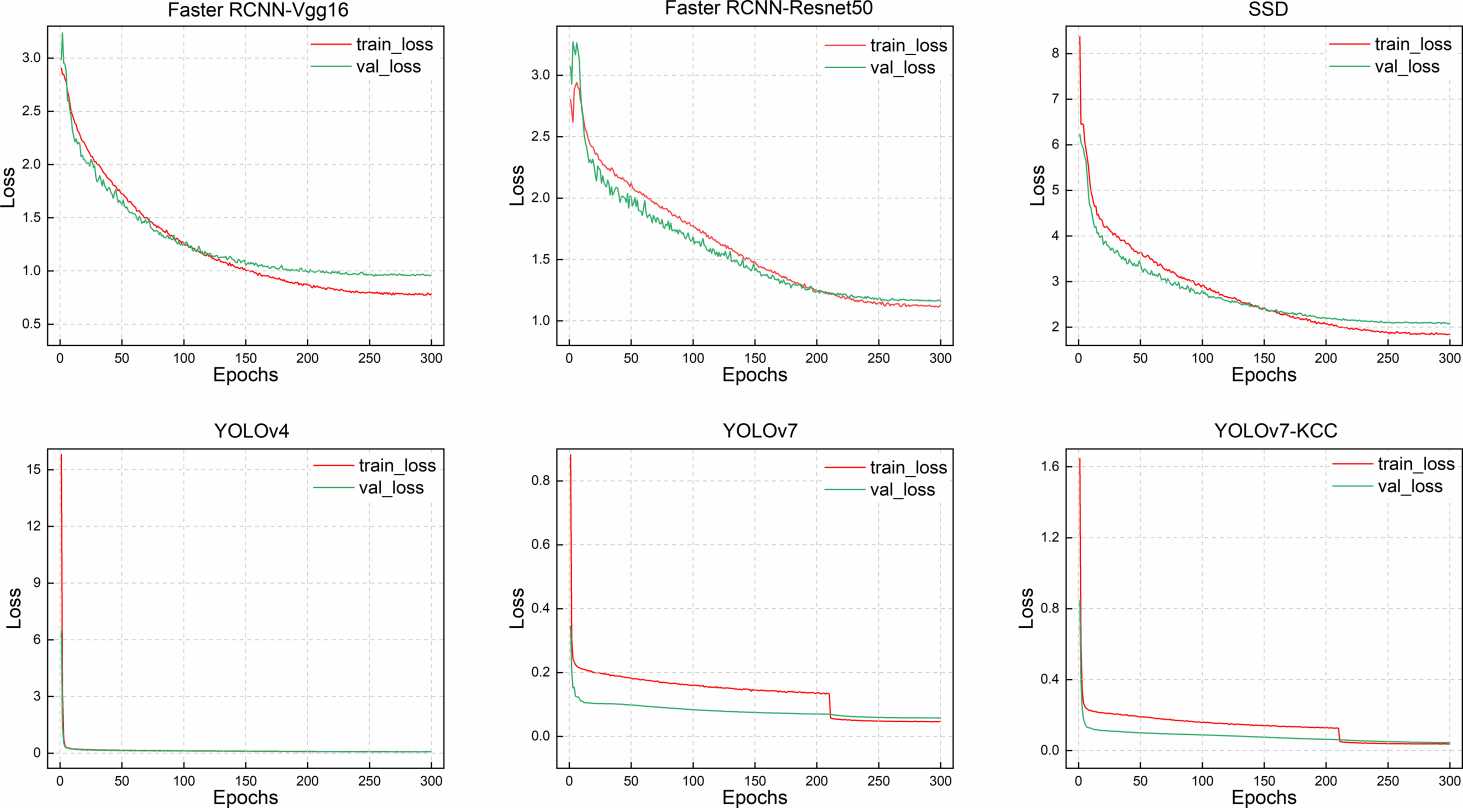
The loss changes of the 6 models.

**Figure 11** illustrates the training loss reduction for these detector models. While SSD approaches the detection accuracy of the YOLOv7 series models after 200 training rounds, its convergence speed is slow with significant early-stage fluctuations. The Faster RCNN-Vgg16 model gradually converges in the training and validation loss curves after the 120th epoch, and by the 270th epoch, it has already converged and no longer varies. However, the loss curve of Faster RCNN-Resnet50 continues to decrease during training, and it only starts to exhibit a convergence trend at the 280th epoch, but still displays a changing trend at the 300th epoch. The model may have architectural issues that prevent it from converging to the optimal solution. This also explains why Faster RCNN-Resnet50 has the lowest accuracy in the training accuracy change graph. Regarding the two-stage detector Faster RCNN, the model has a higher complexity, requiring longer training time and more computing resources, regardless of whether Vgg16 or Resnet50 is used as the backbone network, both having more convolutional layers and parameters. Meanwhile, as a single-stage detector, SSD also has slow convergence speed. After 270 epochs, the region converges, but the training loss still has small fluctuations. Similar to the first two models, the loss curve tends to converge but is not smooth. The improved YOLOv7-KCC model has a significantly faster convergence speed during training than these three models. This is because we added the CBAM attention mechanism to the Neck network layer for feature extraction, enhancing the weight of the object to be detected in both spatial and channel dimensions in the feature distribution, discarding irrelevant feature interference during fitting, and accelerating convergence speed. The YOLOv4 model has a swift loss reduction rate in the early stages of training, and it has already converged after 20 epochs, signifying that the YOLOv4 model can effectively learn data features in the early stages of training and has excellent generalization ability. However, since YOLOv4 uses CSPdarknet53 as the backbone network, the model capacity is insufficient, and the model may not learn enough features, resulting in low training accuracy. The improved YOLOv7-KCC model significantly enhances the model’s feature extraction capability regarding the target object by replacing the ordinary convolution block with the coordinate convolution block in the ELAN multi-branch stacking module. Additionally, the dense residual structure corresponds to so many feature layers, and the residual network is easy to optimize and can improve accuracy by increasing depth. Its internal residual block uses skip connections, which alleviate the gradient vanishing problem caused by increasing depth in deep neural networks.

We provide a comparative analysis of multiple indicators for object detection, as shown in **Table 4**. Firstly, a quantitative analysis of mAP@0.5 is conducted. The detection accuracy of the YOLOv7-KCC model reaches 98.91%, which is 3.69% higher than the original YOLOv7 model’s 95.22%, and 5.97% higher than the SSD model, which is also a single-stage object detection method. Additionally, the YOLOv7-KCC model is not inferior on the strict mAP@[0.5:0.95] indicator, achieving 0.781. mAP@[0.5:0.95] represents the average AP value at different IoU thresholds. From the table, it can be seen that the higher complexity of the Faster RCNN-Vgg16 model sacrifices time and computing resources for accuracy. Although its average precision reached 93.20%, the GFLOPS (Giga Floating Point Operations Per Second) reached 370.01G, with a parameter size of 521.8MB. This is attributed to its trade-off between time, computational resources, and precision. Observing that the GFLOPS of Faster RCNN-Resnet50 reached 939.36G, it is due to the deeper network structure of ResNet50 compared to VGG16. ResNet50 introduces residual connections, allowing for a deeper network without the issues of vanishing or exploding gradients. However, deeper networks typically require more computations. Residual connections introduce additional addition operations, increasing the computational complexity of each residual block. Therefore, in practical applications, the choice of backbone network needs to consider the trade-off between model performance and computational resources. Of course, the classification performance of the Faster RCNN-Resnet50 model is far inferior, with an mAP@[0.5:0.95] score of 0.483, while YOLOv7 scored 0.670. We suggest that some of the loss is caused by ground background interference, different tree growth states, and crown overlap and occlusion. F1 can comprehensively evaluate the model’s precision and recall indicators. YOLOv7-KCC achieved a score of 0.98 here, which is nearly 0.056 higher than YOLOv7, 0.118 higher than SSD, and 0.176 higher than Faster RCNN-Vgg16, demonstrating a balanced performance in precision and recall. In summary, the proposed YOLOv7-KCC model has excellent recognition and classification performance and outstanding detection performance for protective forest tree species.

**Table 4:** Comparison experiments of different models under multiple indicators.

Model	Precision	Recall	mAP@0.5	mAP@[0.5:0.95]	GFlops	F1	Parameter
Faster RCNN-VGG16	72.47%	93.23%	93.20%	0.579	370.01	0.814	521.8MB
Faster RCNN-Resnet50	62.09%	89.43%	87.16%	0.483	939.36	0.732	108.3 MB
SSD	85.48%	89.50%	92.94%	0.604	61.21	0.872	92.6 MB
YOLOv4	91.12%	80.79%	91.05%	0.453	59.79	0.852	244.5 MB
YOLOv7	94.94%	90.18%	95.22%	0.670	104.83	0.924	142.4 MB
YOLOv7-KCC	97.93%	98.12%	98.91%	0.781	105.07	0.98	143.7 MB

We chose the Average Precision (AP) metric to reflect the classification performance of various models on different tree species, as shown in **Table 5**. AP is a metric commonly employed in object detection to evaluate the performance of a model on a specific class. It measures the average performance of a model in terms of detection accuracy and recall for a category by calculating the area under the Precision-Recall curve. *Ulmus pumila* and *Elaeagnus angustifolia*, both characterized by large tree crowns and a high number of samples, exhibit similar classification performance between the baseline model YOLOv7 and Faster RCNN-VGG16, SSD. YOLOv7-KCC, demonstrates a significant improvement in the classification performance of these two tree species. *Populus bolleana*, distinguished from other tree species by its upward-extending branches, conical tree shape, and distinct color characteristics, is relatively easily classified. Consequently, models of various types show higher classification AP for *Populus bolleana*. *Haloxylon ammodendron* and dead trees, with fewer samples and less distinctive features, particularly in the case of dead trees characterized by small targets, pose challenges for classification. Through our improvements, YOLOv7-KCC exhibits a notable enhancement in identifying small targets such as dead trees, with a significantly higher classification AP compared to the contrast models.

**Table 5:** AP values of different classification models in five tree species.

Model	Ulmus pumila	Populus bolleana	Haloxylon ammodendron	Elaeagnus angustifolia	dead trees
Faster RCNN-VGG16	92.18%	96.92%	96.39%	90.54%	89.97%
Faster RCNN-Resnet50	84.59%	93.51%	93.28%	82.98%	81.42%
SSD	92.26%	96.9%	95.7%	91.93%	87.91%
YOLOv4	89.05%	96.04%	93.94%	90.32%	85.89%
YOLOv7	92.97%	98.6%	96.42%	94.18%	93.91%
YOLOv7-KCC	99.74%	99.97%	96.69%	99.78%	98.38%

**Figure 12:**
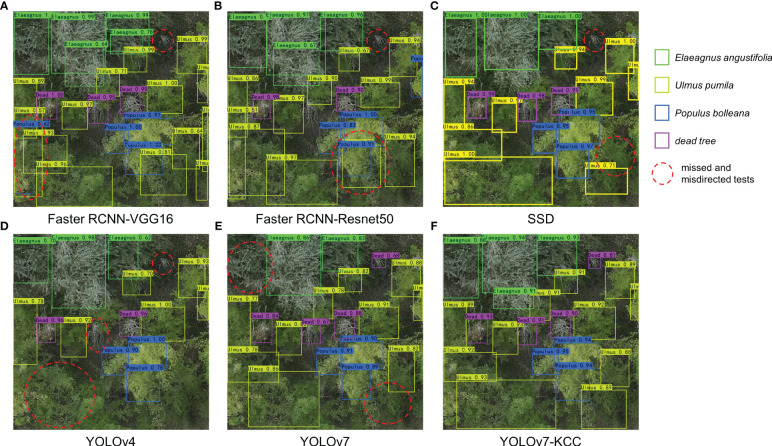
Test results of the 6 models. **(A-F)** represent the results of Faster RCNN-VGG16, Faster RCNN-Resnet50, SSD, YOLOv4, YOLOv7, and YOLOv7-KCC, respectively.

To better understand the performance of the model, **Figure 12** demonstrates the detection results of six models on randomly selected images from the test set. As shown in the figure, both YOLOv7 and the improved YOLOv7-KCC model have higher recognition capabilities for small targets such as dead trees in the orthographic tree crown images compared to other models. Faster RCNN-VGG16 and Faster RCNN-Resnet50 have both shown false detection and confusion, classifying *Ulmus pumila* crowns as *Ulmus pumila* and then as *Populus bolleana*. This indicates that these models have weaker recognition capabilities for tree species with similar color features, and are therefore not suitable for detecting this type of dataset. Our improved YOLOv7-KCC model has increased its confidence in detecting targets by adding an attention mechanism, which optimizes the features of different targets in the image and discards irrelevant information. There are more missed detections in the SSD and YOLOv4 models, and the YOLOv7 model also has some undetected targets. We have highlighted these targets with red dashed circles in the figure, with YOLOv4 having the most significant missed detections. The improved YOLOv7-KCC model replaces the backbone network convolution module with CoordConv convolution, which inputs the coordinate information as an extra channel in the convolution operation, allowing the model to learn more precise position information when processing images. This improves the model’s ability to perceive and understand target location information, thereby improving the accuracy and robustness of target detection. Additionally, another advantage of CoordConv convolution is that it reduces the model’s dependence on position information, thereby improving its generalization ability. The YOLOv7-KCC model did not exhibit any false detection or confusion, nor did it miss multiple targets. Therefore, the YOLOv7-KCC model is highly suitable for the protective forest tree species classification in this study.

## 5 Discussion

In this paper, we employed geometric transformations and color transformations (rotation, mirroring, addition of Gaussian noise, and contrast adjustment) as two data augmentation methods to process the data. These augmentation techniques augment the sample size, thereby elevating the model’s generalization capacity, mitigating the risk of overfitting, and enhancing the model’s robustness. The K-means++ algorithm was adopted to cluster anchor boxes in the tree species dataset, its can elevate both the training velocity and precision of the model. Experimental findings reveal that subsequent to implementing the K-means++ algorithm for dataset clustering, the model’s mAP@0.5 has ascended by 1.23% in comparison to the baseline YOLOv7 model. We have refined the original YOLOv7 model, substituting certain convolutional layers in the main network with CoordConv. This integration incorporates coordinate information as supplementary features, fortifying the model’s feature extraction capabilities. Experimental results indicate an improvement in recognition accuracy following the enhancement of the main network. To address issues arising from complex backgrounds and crown overlap, we introduced the CBAM attention mechanism into the Neck network. This augmentation bolsters the model’s perceptual capabilities towards features, suppressing noise and irrelevant information in the images, thereby enhancing model performance. The three effective feature maps extracted from the main network are subjected to the CBAM module, enabling the learning of channel correlations and importance, followed by convolutional operations on the weighted processed feature maps. This approach resolves challenges related to crown obscuration and ambiguous delineation in the tree species layer within the dataset. Upon evaluation on the test dataset, our YOLOv7-KCC model has demonstrated exceptional performance compared to five other object detection models. Its mAP@0.5 reached 98.91%, surpassing Faster RCNN-VGG16, Faster RCNN-Resnet50, SSD, YOLOv4, and YOLOv7 by 5.71%, 11.75%, 5.97%, 7.86%, and 3.69%, respectively. In terms of mAP@[0.5:0.95], the improved model achieved 0.781, an improvement of approximately 0.111 over YOLOv7, and exhibited a nearly 5.6% improvement in the F1 score, effectively balancing precision and recall. Additionally, our model significantly reduces the parameter count compared to Faster RCNN-VGG16 and YOLOv4 models, with minimal differences from other models. We aim to investigate the deployment of lightweight models for real-time detection on UAVs.

## 6 Conclusion

In general, an improved YOLOv7_Kmeans++_CoordConv_CBAM (YOLOv7- KCC) model based on YOLOv7 is proposed for tree species classification in shelterbelts. Firstly, we constructed a dataset for protective forests, augmenting its sample size through geometric and color transformations, thereby mitigating the risk of overfitting and enhancing the model’s generalization capability. Second, we substituted the conventional convolution modules with CoordConv convolution modules to acquire supplementary coordinate information, facilitating more precise prediction of target positions across diverse scenarios, thereby elevating detection accuracy and averting instances of omission or misjudgment. Finally, by introducing an attention mechanism, we incorporated CBAM attention modules into the feature extraction and fusion processes, considering both channel and spatial dimensions. This module adeptly captures local details and global contextual information for effectively suppressing irrelevant features, thereby enhancing the model’s capacity to discern crucial features. Experimental outcomes demonstrate the outstanding performance of our proposed methodology in terms of classification accuracy, rendering it effectively deployable on intelligent terminals for the classification of protective forest tree species. Moreover, our research furnishes theoretical insights for the classification of tree species in other regions and research domains.

## Data availability statement

The raw data supporting the conclusions of this article will be made available by the authors, without undue reservation.

## Author contributions

YL: Conceptualization, Software, Writing – original draft, Writing – review & editing. QZ: Funding acquisition, Supervision, Writing – review & editing. XW: Methodology, Supervision, Writing – review & editing. YS: Data curation, Validation, Writing – review & editing. WT: Data curation, Writing – review & editing. YR: Writing – review & editing.
